# Association Between Turning Mobility and Cognitive Function in Chronic Poststroke

**DOI:** 10.3389/fneur.2022.772377

**Published:** 2022-02-23

**Authors:** Yi-Chun Kuan, Li-Fong Lin, Chien-Yung Wang, Chia-Chen Hu, Pei-Jung Liang, Shu-Chun Lee

**Affiliations:** ^1^Department of Neurology, School of Medicine, College of Medicine, Taipei Medical University, Taipei, Taiwan; ^2^Department of Neurology, Taipei Medical University Shuang Ho Hospital, New Taipei City, Taiwan; ^3^School of Gerontology Health Management, College of Nursing, Taipei Medical University, Taipei, Taiwan; ^4^Department of Physical Medicine and Rehabilitation, Taipei Medical University-Shuang-Ho Hospital, New Taipei City, Taiwan; ^5^Department of Physical Medicine and Rehabilitation, Taipei Medical University-Wan Fang Hospital, Taipei, Taiwan; ^6^Division of Physical Therapy, Department of Physical Medicine and Rehabilitation, Taipei Medical University Hospital, Taipei, Taiwan; ^7^Department of Rehabilitation Medicine, Taipei Tzu Chi Hospital, Buddhist Tzu Chi Medical Foundation, New Taipei City, Taiwan

**Keywords:** cognitive domains, cognitive function, stroke, turning mobility, wearable sensors

## Abstract

Turning difficulties are common in patients with stroke. The detrimental effects of dual tasks on turning indicate a correlation between turning and cognition. Cognitive impairment is prevalent after stroke, and stroke patients with mild cognitive impairment had a poorer turning performance than did stroke patients with intact cognitive abilities. Therefore, we investigated the association between turning mobility and cognitive function in patients with chronic poststroke. Ninety patients with chronic stroke (>6 months post-stroke) were recruited. Angular velocity was assessed using wearable sensors during 180° walking turns and 360° turning on the spot from both sides. Global cognition and distinct cognitive domains were assessed using the Mini-Mental State Examination. In patients with stroke, turning mobility was significantly associated with global cognitive function and distinct cognitive domains, such as visuospatial ability and language. The balance function and lower limbs strength were mediators of the association between cognition and turning. The association highlights the complexity of the turning movement and dynamic motor and cognitive coordination necessary to safely complete a turn. However, our findings should be regarded as preliminary, and a thorough neuropsychological assessment to provide a valid description of distinct cognitive domains is required.

## Introduction

The turning mobility frequently causes falls in patients with stroke (1). The incidences of hip fractures caused by falls that occurred while turning is 8 times higher than that occurring while walking (2). More than 40% of walking involves making turns (3). Thus, turning safely is crucial for maintaining independence in the activities of daily living. Numerous studies have revealed that, compared with age-matched healthy controls, patients with stroke require a longer time and more steps to turn (4–6). Furthermore, patients with stroke covered a longer distance while turning than their healthy counterparts and also exhibited a different trajectory for their center of gravity (7). Their center of gravity moves at a slower speed and is maintained at the base of support of the body during turning (8). Their body segments exhibit the *en bloc* turn phenomenon when turning while walking, indicating instability during turning (9). Thus, patients with stroke have substantially more difficulties in turning than normal adults.

Research on the effects of dual tasks on turning in patients with stroke was the first to identify a correlation between turning and cognition. Hollands and colleagues revealed that patients with stroke demonstrated a longer turn time, greater step width, and longer single limb support phase when turning 90° while walking and performing arithmetic tasks than while performing only a turning task, indicating that two tasks interfere with each other and both tasks are assumed to compete for the same cognitive resources in the brain (10). Manaf et al. conducted a full-body kinematic analysis and reported that patients with stroke had earlier axial segment reorientation latency with respect to the turn onset while performing a dual-cognitive task (a counting backward task during turning) than while performing a single task (only a turning task) and a dual-motor task (holding a glass of water during turning) (11). Cognitive interference requires increased attentional resources and therefore generates a greater dual-task interference, greatly affecting turning.

Recent evidence has further shown that turns are associated with processing speed and executive function in healthy adults (12), and correlate with attention (13), and visuospatial ability (14) in patients with Parkinson's disease. Attentional demands might be required when performing a challenging motor task such as turning. Processing of different visuospatial and afferent inputs might also necessary to enable clear directional movement. These cognitive domains direct higher-order cognitive control of gait and posture, and are responsible for some levels of planning, organization, and orientation in space. However, this has not been investigated in patients with stroke. Cognitive impairment is prevalent after stroke, and approximately 80% of patients exhibit impairment in at least one cognitive domain (15). Impairments were found most frequently in memory, visuospatial and executive functions, which could be an important contributor to turning dysfunction in patients with stroke (15). Stroke patients with mild cognitive impairment have been reported to have a longer time to turn around in the timed up and go (TUG) test than did stroke patients with intact cognitive abilities (16, 17). Stroke combined with cognitive decline may have a greater influence on turning performance than stroke itself (16, 17).

Previous studies investigated the correlation of cognition and turning but the majority focused on turning while walking. None of studies compared the differences between turning while walking and turning on the spot in terms of the cognitive demands. Investigating different turning tasks and turning angles may be needed because various turning tasks may have different motor programming and turns at different angles are executed during daily activities. Falling is one of the most common complications of stroke patients and turning is an activity that frequently causes falls. However, turning has only been explored in recent years compared with the investigation on straight walking. It is essential for improving our understanding of turning mobility among stroke patients. Physical functions such as muscle strength, motor recovery in the lower limbs, functional balance, and walking capacity (6, 18, 19), have been reported to associate with turning, cognition may also be a contributor to turning difficulties in stroke patients. Therefore, this study investigated the association between turning mobility and cognitive function in patients with chronic poststroke.

## Materials and Methods

### Participants

This cross-sectional observational study was conducted from October 2019 to January 2021 at Shuang-Ho Hospital, Wan Fang Hospital, Taipei Medical University Hospital, and Taipei Tzu Chi Hospital in Taipei and New Taipei city, Taiwan. The inclusion criteria were (1) age 20 to 99 years, (2) survivors of a single unilateral stroke with hemiparesis for at least 6 months before recruitment to the study, (3) ability to walk >10 m independently, and (4) ability to provide informed consent and follow oral command. Patients meeting the following criteria were excluded: (1) additional musculoskeletal conditions or hemineglect that could affect the evaluation and (2) dementia or aphasia that could prevent participants from following instructions. All participants had undergone medical treatment and rehabilitation before the study and had stable stroke conditions throughout the study. All eligible participants provided written informed consent before their participation in the study, which was conducted in accordance with the Declaration of Helsinki, and approved by the Institutional Review Board of Taipei Tzu Chi Hospital, Buddhist Tzu Chi Medical Foundation (Reference No. 08-XD-051), and Taipei Medical University Joint Institutional Review Board (N201912127).

### Procedures

Demographic data, namely age, sex, and body mass index; medical history (stroke type and lesion side); poststroke duration; and walking device use were extracted from the medical record of patients with stroke, and their physical function was examined using the Berg Balance Scale (BSS; for lower limb balance) and five times sit-to-stand (FTSTS; for lower limb strength). The BBS is a reliable and valid measure for people with stroke (20), and it is composed of 14 balance-related tasks individually scored from 0 (inability to perform task) to 4 (independent ability to perform task). The highest total score is 56, which indicates the optimal balance function. In individuals with stroke, scores of 0 to 20 represent balance impairment, of 21 to 40 represent acceptable balance, and of 41 to 56 represent good balance. The FTSTS test was reported to be reliable and valid in patients with stroke (21). Participants were seated on a 45-cm-high standard chair without armrests and instructed to perform the sit-to-stand motion as rapidly as possible 5 times. The time to complete the task was recorded, with a cutoff value of longer than 12 s for poor lower limb strength Finally, the turning performance and cognitive function of all participants were evaluated. All assessments were conducted individually in the laboratory of the hospital within 1 h by a well-trained research assistant with a health care–related background.

### Turning Performance

Turning performance was measured using APDM Opal wireless sensors and Mobility Lab software (APDM, Portland, OR, USA). The Opal is a lightweight (22 g) inertial sensor with a battery life of 16 h and 8 GB of storage. Three Opal inertial sensors were attached to the participant by using Velcro elastic bands, with one on the middle lower back (fifth lumbar vertebra process) and one on the top of each foot. Data were recorded at 128 Hz, stored in the internal memory of the Opal sensor, and subsequently uploaded to a personal computer for offline analysis. The data were exported directly as reported from the APDM system.

Participants were instructed to perform 2 turning tasks [180° walking turns (4) and 360° turn on the spot (6)] at a self-selected pace. Turning 180° while walking is commonly assessed using the TUG test (22), and turning 360° on the spot is one of the items in the BBS assessment (23) and Tinetti motor assessment (24). Before the tests, the researcher demonstrated the procedure to the participants. All participants performed a practice trial to familiarize themselves with the test before the 2 actual trials. Participants wore their regular footwear during the tests. The researcher noted the direction in which the participants opted to turn and asked them to repeat the procedure in the opposite direction.

The angular velocity (°/s) of both 180° and 360° turns were recorded for the analysis; angular velocity represents the mean angular velocity of the trunk along the rotation axis during turning, and decreased angular velocity indicates increased instability although there has been no normative value reported previously (25). This parameter was selected for the study because our previous research indicated that the turning velocity may be more sensitive than the time duration and number of steps required for representing the quality of the turning performance (26). The horizontal rotational rate of the lumbar sensor was used with a minimum of 45° accompanied by at least one right and one left foot stepping to detect turns. Humans find it challenging to make more than a slight turn in < 0.5 s or to complete an extremely slow turn in >10 s while walking. Therefore, only turns within a duration of 0.5 to 10 s and turn angles of >45° were considered (27). The algorithm for detecting and characterizing turning has been detailed previously (27, 28).

### Cognitive Function

To assess cognitive function, we used the Mini-Mental State Examination (MMSE), which is a 30-point questionnaire extensively used in clinical and research settings. The MMSE is a reliable and valid measure for research in people with stroke (29). It is composed of 5 cognitive domains and 11 individual items. The 5 domain are as follows: (1) Orientation: temporal orientation (5 points) and spatial orientation (5 points); (2) Memory: immediate memory (3 points) and delayed recall (3 points); (3) Attention: serial subtraction (5 points); (4) Language: naming (2 points), verbal repetition (1 points), reading (1 points) and writing (1 points) a sentence, and verbal comprehension (3 points); and (5) Visuospatial ability: construction (1 points). Any score of 26 or more (out of 30) indicates a normal cognition. Below this, scores can indicate severe (≤9 points), moderate (10–19 points) or mild (20–25 points) cognitive impairment.

### Statistical Analysis

Statistical analyses were performed using SPSS version 19.0 (SPSS, Chicago, IL, USA). The significance level was set to *p* < 0.05. To analyze whether any correlation between participants' characteristics, cognitive function, and turning performance, the Spearman's rank correlation test and Mann-Whitney U test was used. Any significant correlations among cognition, participants' characteristics and turning tasks were found, linear regressions were conducted in three paths (between cognition and turning, between cognition and participants' characteristics, and between participants' characteristics and turning) in order to assess the potential mediator effect (participants' characteristics) on the association between cognition and turning.

## Results

In total, 90 patients with stroke were recruited for this study (Table 1). The mean age of participants are around 60 years old with the majority are men. The mean body mass index is borderline overweight, and more than half of them use assistive devices in their daily life. Nearly 70% of participants are ischemic stroke while 30% are hemorrhagic stroke. Participants are almost equally divided between right and left hemisphere damage. Their mean MMSE score is 26, indicating a normal cognition. In terms of physical function, their mean score of 45 on BBS represents good balance and mean time of 22s on FTSTS represents poor lower limbs strength.

**Table 1:** Demographic characteristics, cognitive function and turning performance of patients with stroke (*N* = 90).

**Participants' characteristics**	
Age (years)	59.40 ± 10.53 (35–93)
Sex (male, *n*, %)	61 (68%)
Body mass index (kg/m^2^)	24.64 ± 3.82 (16.02–37.64)
Lesion side (right, *n*, %)	46 (51%)
Post-stroke duration (month)	42.73 ± 46.47 (6–207)
Lesion type- Infarction (*n*, %)	60 (67%)
Lesion type- Hemorrhage (*n*, %)	30 (33%)
Assistant devices (*n*, %)	49 (54%)
Five Timed Sit-to-Stand (s)	22.35 ± 14.03 (5.91–109.00)
Berg Balance Scale (score/56)	44.74 ± 7.67 (19–56)
**Cognitive function**	
Mini-Mental State Examination Score (score/30)	26.93 ± 2.91 (16–30)
Orientation (score/10)	9.53 ± 1.56 (0–10)
Memory (score/6)	5.26 ± 0.82 (3–6)
Attention (scor /5)	4.35 ± 0.97 (1–5)
Language (score/8)	7.13 ± 1.09 (3–8)
Visuospatial (score/1)	0.72 ± 0.45 (0–1)
**Turning performance**	
180° turns toward paretic side (°/s)	119.59 ± 36.31
360° turns toward non-paretic side (°/s)	127.87 ± 39.27
180° turns toward paretic side (°/s)	127.16 ± 45.77
360° turns toward non-paretic side (°/s)	139.61 ± 50.72

*Data are presented as mean ± standard deviation (min-max) and number (percentage)*.

The MMSE total score was significantly associated with all turning tasks except 360° turns to the non-paretic side (Table 2). In terms of cognitive domains, only visuospatial ability was significantly associated with all turning tasks while language was associated with all turning tasks except 360° turns to the non-paretic side. Orientation, memory, and attention were not associated with turns. On the top of that, MMSE score, language and visuospatial ability were significantly correlated with FTSTS and BBS. The FTSTS and BBS were also significantly correlated with all turning tasks. Due to significant correlations among cognition (MMSE, language and visuospatial ability), participants' characteristics (FTSTS and BBS) and turning tasks, further mediator analysis was conducted (Table 3). The results showed that FTSTS and BBS were mediators of the association between MMSE and turning tasks (180° and 360° turns to the paretic side). The FTSTS and BBS also mediated the association of language and turning tasks (180° and 360° turns to the paretic side). However, only FTSTS was found as a mediator of the association between visuospatial and all turning tasks except 360° turns to the non-paretic side.

**Table 2:** Correlation analysis between cognitive function, turning performance and participants' characteristics in patients with stroke.

	**180° turns**		**360° turns**		**Participants' characteristics**								
	**Toward P side**	**Toward NP side**	**Toward P side**	**Toward NP side**	**Age**	**Sex**	**BMI**	**Lesion side**	**Post-stroke duration**	**Lesion type**	**Assistive devices**	**FTSTS**	**BBS**
**Cognition**													
MMSE score	**0.272**	**0.275**	**0.247**	0.194	−0.128	815.5	0.130	733.5	−0.165	826.5	919.0	**−0.281**	**−0.317**
Orientation	−0.130	−0.049	−0.097	−0.079	0.016	743.5	−0.038	908.0	−0.115	817.0	926.5	−0.125	0.090
Memory	0.019	0.099	−0.003	−0.053	0.008	816.5	0.240	777.5	−0.118	776.5	955.5	−0.034	**0.261**
Attention	−0.041	−0.083	−0.027	−0.065	−0.161	869.5	0.214	968.0	−0.119	655.5	934.5	−0.104	0.185
Language	**0.284**	**0.276**	**0.217**	0.164	−0.075	844.5	0.047	866.5	−0.085	753.5	954.0	**−0.245**	**0.350**
Visuospatial	**0.338**	**0.247**	**0.258**	**0.274**	−0.149	863.5	0.089	917.0	−0.019	797.5	961.5	**−0.299**	**0.209**
**Characteristics**													
Age	−0.191	−0.167	−0.181	−0.180									
Sex	774.5	811.0	756.0	738.0									
BMI	−0.057	−0.096	−0.099	−0.139									
Lesion side	943.0	928.0	966.5	888.0									
Duration	−0.009	0.022	0.029	0.066									
Type	759.5	802.0	794.0	706.0									
Devices	760.0	880.0	768.0	901.5									
FTSTS	**−0.589**	**−0.571**	**−0.617**	**−0.624**									
BBS	**0.621**	**0.560**	**0.663**	**0.539**									

*Data are presented as r values except sex, lesion side, lesion type and assistive devices presented as U values. Bold font indicates statistical significance at p < 0.05. P, paretic; NP, non-paretic; BMI, body mass index; MMSE, mini mental state examination; FTSTS, five times sit-to-stand; BBS, berg balance scale*.

**Table 3:** Mediator effect of FTSTS and BBS on the association between cognition and turning in patients with stroke.

	**180° turns**				**360° turns**				**Participants' characteristics**			
	**Toward P side**		**Toward NP side**		**Toward P side**		**Toward NP side**		**FTSTS**		**BBS**	
	**B (SE)**	***p*** **value**	**B (SE)**	***p*** **value**	**B (SE)**	***p*** **value**	**B (SE)**	***p*** **value**	**B (SE)**	***p*** **value**	**B (SE)**	***p*** **value**
**Cognition**												
MMSE score	2.742	**0.041**	2.548	0.081	3.797	**0.029**	1.744	0.397	−0.796	**0.020**	0.739	**0.004**
Language	18.403	**0.033**	13.088	0.163	30.706	**0.004**	22.537	0.060	−8.883	**0.005**	6.094	**<0.001**
Visuospatial	10.890	**0.005**	10.903	**0.008**	9.574	**0.047**	9.342	0.072	−4.572	**0.001**	1.333	0.072
**Participants' characteristics**												
FTSTS	−2.006	**<0.001**	−2.050	**<0.001**	−2.767	**<0.001**	−2.505	**<0.001**				
BBS	3.106	**<0.001**	3.016	**<0.001**	4.226	**<0.001**	3.927	**<0.001**				

*B, unstandardized parameter estimation; SE, standard error for B. Bold font indicates statistical significance at p < 0.05. P, paretic; NP, non-paretic; MMSE, mini mental state examination; FTSTS, five timed sit-to-stand; BBS, berg balance scale*.

## Discussion

This is the first study to analyze the association between turning mobility and cognitive function after stroke. Our findings indicate that turning mobility is significantly associated with global cognitive function and distinct cognitive domains, such as visuospatial ability and language, in patients with stroke. Mediator analysis revealed that balance function and lower limbs strength played a mediating role in the relationship between cognitive function and turning mobility.

The correlation between turning mobility and global cognition has been observed among patients with stroke in the current study, which was line with previous studies (13, 30, 31). Studies have indicated a negative effect of dual-tasking on turning performance (10, 11), and the detrimental effect was amplified in patients with poorer cognition (16, 17), which may be due to limited cognitive capacity (32). When a task is challenging, it imposes additional cognitive demands. For patients with stroke having a limited cognitive capacity because of brain injury, turning is a complex form of walking that is more cognitively demanding than straight walking. Such cognitive–motor interference or inappropriate use of limited cognitive resources causes an exacerbation of motor impairments. In fact, the role of cognition on turning has been supported by some studies, which have reported an association between higher prefrontal cortex activity and poorer turning performance in older people (30) and individuals with neurological disorders (14). Prefrontal cortex activity increased during the transition from straight walking to turning, indicating that the prefrontal cognitive control could compensate for motor deficits (33). Turning seems to be less autonomous than is walking in a straight line because it involves more interlimb coordination, more coupling between posture and gait, and modifications of locomotor patterns, requiring a high cortical control that plays a crucial role in postural transitions.

Our study found that turning is associated with distinct cognitive domains. Visuospatial ability was observed to be associated with turning, which is in line with previous studies (34, 35). Turning might place excessive demands on visuospatial processing to enable the directional movements required for accomplishing a change in direction while walking. Several studies have proposed a visuospatial contribution to gait, particularly gait stability, in older adults (36) and patients with Parkinson's disease (37). Such individuals rely on visual information for control of balance and locomotion and adjust their limb and axial motor control through visual feedback, which are the elements for the successful completion of the turning task. We also found an association with language, which was not reported previously. In fact, research has demonstrated language to be associated with gait speed in studies on walking and cognition (38, 39). The cerebral region, such as Broca's area, is involved in sentence processing (40). An imaging study reported a correlation of gait disorder with activation of the contralateral inferior frontal cortex (Broca's area), contralateral sensory motor cortex, and homolateral cerebellum. Neuroanatomical evidence reveals a direct connection between Broca's area and the supplementary motor area (41). We posit that Broca's area facilitates walking during an alteration of gait control, such as turning. However, this explanation is speculative and should be empirically evaluated.

Such correlations were not found in the remaining distinct cognitive functions in the current study, although attention (12), processing speed (35), and executive function (12) have been reported to be correlated with turning in previous studies. This disparity may be attributed the attention domain of the MMSE focusing only on an item of serial subtraction, may not adequately represent the attention function to detect associations. Additionally, MMSE does not contain the cognitive domains of processing speed and executive function for analyzing their relevance to turning, and thus their correlations remain unclear.

One of the most widely used tools for cognition evaluation is the MMSE, which has been validated and extensively used in both clinical practice and research. Despite its widespread use, whether the scores on individual items and domains of the MMSE can represent the cognitive domain remains uncertain. Although some studies have concluded that subtests were domain specific (42, 43), a study indicated that a part of the subtests lack sufficient validity to warrant a conclusion of their domain specificity (44). Thus, a thorough neuropsychological assessment to provide a valid description of an individual's cognitive profile is required for future studies. For instance, the Digit Span Forward and Trail Making Test A are commonly used for attention and processing speed assessments; the Digit Span Backwards can be used to assess working memory, and the Trail Making Test B for executive function. Impairments in patients with stroke are most frequently found in memory and visuospatial and executive functions (15), which should be examined preferentially to justify their relationship to turning. Our findings should be considered preliminary.

Lower limbs strength and balance function were introduced as the mediators of the association between cognition and turning in the current study, suggesting that cognition affects muscle strength as well as balance and subsequently results in poor turning performance. Previous studies have shown that the lower limbs strength and balance control correlated with cognitive function (45, 46) and both also contributed to turning difficulties (18, 19). Motor and cognitive deficits commonly interact through cognitive–motor interference, and it is therefore to be expected that strength and balance played a mediating role in the relationship between cognition and turning.

It is also worth mentioning that MMSE score and language function were correlated with all turning tasks except 360° turns to the non-paretic side. The correlations were observed in specific turning situations only. Turning while walking may be more difficult to execute than turning on the spot because it is affected by impaired motor planning and patients with stroke have difficulty in changing from one motor program (walking) to another (turning). Also, turning to the paretic side was more challenging than turning to the other side (26) and associated with instability and falls (2). However, visuospatial ability was significantly correlated with all turning tasks. Steering is an essential component of goal-directed locomotion, allowing individuals to walk toward the desired direction while avoiding static or dynamic obstacles along the travel path (9). Stroke patients with poorer cognition or impairments in language or visuospatial ability may be more prone to instability when performing walking turns or turning to the paretic side, significantly elevated fall risks. Such findings provide insight into the effects of cognitive factors in falls risk for specific turning situations.

Once the association between cognition and turning after stroke is established, turning mobility can be used to further enhance the prediction of cognitive decline in the stroke population. Approximately 70% of patients with stroke have cognitive impairment in the first year after the stroke (47). The prevalence of cognitive impairment after a stroke is high and may progress to dementia, which affects secondary prevention, rehabilitation, prognosis, and quality of life (48). Studies have revealed that the BBS and 10-m walk test could predict cognitive impairment in a year after stroke (49), indicating that motor biomarkers such as balance and gait can be used for early detection of cognitive impairment. However, a balance test battery includes multiple test items, and a walking test applies to ambulatory poststroke only. Assessment of turning is comparatively simple and quick to administer, which may specifically be suitable for those who walk with difficulty or are unable to walk for a long distance.

Relative to studies that have investigated turning in patients with stroke, interventional studies aimed at improving turning performance remain scarce. Our findings of a significant association between turning and cognition indicate that interventional studies could possibly incorporate cognitive training into the turning exercise. The integration physical and cognitive exercise into training seems to render more favorable results in both physical and cognitive performance than when either type of training is used alone in many populations, including those with stroke (50, 51), because of the enhancement of resting-state functional connectivity between the medial prefrontal cortex and medial temporal lobe regions (52). Turning performance could potentially be improved if turning training is combined with cognitive training, and such improvement may be related to the improvement of specific cognitive functions related to turning.

The strength of the current study is that 90 participants with poststroke from 4 hospitals were enrolled. Thus, problems associated with the use of a small sample size and heterogeneous sample were absent. Furthermore, 2 turning tasks, 180° walking turns and 360° turning on the spot, conducted in the present study eliminated bias caused by assessment of different turning tasks or different turning angles. Turning performance could vary in terms of turning tasks and turning angles. Various turning tasks may involve distinct motor programming, and turns at different angles are executed during daily activities.

A few limitations of this study can serve as guidance for follow-up studies. First, our participants obtained a mean MMSE score of 26.93 ± 2.91 (range: 16–30), they did not have dementia, and they were able to provide informed consent and follow instructions; thus, our sample may not be completely representative of this population. Our results can likely only be generalized to high-functioning patients with stroke. Studies with more participants with moderate or severe cognitive impairments should be conducted in future to improve the generalizability of the findings and strengthen the correlation of distinct cognition and turns. Second, a study revealed that natural turns in the home can be used to efficiently differentiate between those who fall recurrently from those who have not fallen; however, prescribed turns in the laboratory cannot differentiate between older adults with and without a history of falls (34). Thus, laboratory-based turning measurement may not adequately reflect real-life functioning. The laboratory environment is static, and the vigilance of the researcher reduces anxiety and fear of falling, which could temporarily enhance the participant's performance and unintentionally mask turning difficulty. The lack of significant associations of certain cognitive domains with turning mobility may be because these turns were all prescribed movements evaluated in a laboratory. Third, neither visual acuity nor use of corrective vision devices were measured and recorded in the study. Poor visual function could possibly influence visuospatial ability and execution of movement. However, all participants can read and sign the consent forms and they are encouraged to wear spectacles to get the better eyesight during the testing, which may reduce the impact. Finally, our findings indicated a significant relationship between turning parameters and cognitive function; however, the strength of the correlation was low. Thus, cognitive functions could be one of the several factors affecting turning performance.

## Conclusions

This is the first study to analyze the association between turning mobility and cognitive function after stroke. Our findings showed that turning mobility was significantly associated with global cognitive function and distinct cognitive domains, such as visuospatial ability and language, in patients with stroke. The association between turning and cognition highlights the complexity of turning and the dynamic motor and cognitive coordination necessary to safely execute a turn. However, our findings should be regarded as preliminary, and a thorough neuropsychological assessment is essential to establish a robust association between turning mobility and distinct cognitive domains.

## Data Availability Statement

The raw data supporting the conclusions of this article will be made available by the authors, without undue reservation.

## Ethics Statement

The study was approved by the Institutional Review Board of Taipei Tzu Chi Hospital, Buddhist Tzu Chi Medical Foundation (Reference No. 08-XD-051) and Taipei Medical University Joint Institutional Review Board (N201912127). The patients/participants provided their written informed consent to participate in this study.

## Author Contributions

S-CL was a major contributor in study design. Y-CK, L-FL, C-YW, C-CH, and P-JL have done the data collection. S-CL and Y-CK have done the manuscript writing and analyzed and interpreted data. All authors read and approved the final manuscript.

## Funding

This work was supported by the Taiwan Ministry of Science and Technology (Grant No. MOST 108-2314-B-038-031-MY3).

## Conflict of Interest

The authors declare that the research was conducted in the absence of any commercial or financial relationships that could be construed as a potential conflict of interest.

## Publisher's Note

All claims expressed in this article are solely those of the authors and do not necessarily represent those of their affiliated organizations, or those of the publisher, the editors and the reviewers. Any product that may be evaluated in this article, or claim that may be made by its manufacturer, is not guaranteed or endorsed by the publisher.
